# Template-controlled mineralization: Determining film granularity and structure by surface functionality patterns

**DOI:** 10.3762/bjnano.6.180

**Published:** 2015-08-20

**Authors:** Nina J Blumenstein, Jonathan Berson, Stefan Walheim, Petia Atanasova, Johannes Baier, Joachim Bill, Thomas Schimmel

**Affiliations:** 1 Institute for Materials Science, University of Stuttgart, Heisenbergstraße 3, D-70569 Stuttgart, Germany; 2 Institute of Nanotechnology, Karlsruhe Institute of Technology (KIT), Hermann-von-Helmholtz-Platz 1, Eggenstein-Leopoldshafen, D-76344, Germany; 3 Institute of Applied Physics and Center for Functional Nanostructures, Karlsruhe Institute of Technology (KIT), Wolfgang-Gaede-Strasse 1, D-76131 Karlsruhe, Germany

**Keywords:** bioinspired synthesis, polymer-blend lithography, surface functionality, template-controlled self-assembly, zinc oxide thin film

## Abstract

We present a promising first example towards controlling the properties of a self-assembling mineral film by means of the functionality and polarity of a substrate template. In the presented case, a zinc oxide film is deposited by chemical bath deposition on a nearly topography-free template structure composed of a pattern of two self-assembled monolayers with different chemical functionality. We demonstrate the template-modulated morphological properties of the growing film, as the surface functionality dictates the granularity of the growing film. This, in turn, is a key property influencing other film properties such as conductivity, piezoelectric activity and the mechanical properties. A very pronounced contrast is observed between areas with an underlying fluorinated, low energy template surface, showing a much more (almost two orders of magnitude) coarse-grained film with a typical agglomerate size of around 75 nm. In contrast, amino-functionalized surface areas induce the growth of a very smooth, fine-grained surface with a roughness of around 1 nm. The observed influence of the template on the resulting clear contrast in morphology of the growing film could be explained by a contrast in surface adhesion energies and surface diffusion rates of the nanoparticles, which nucleate in solution and subsequently deposit on the functionalized substrate.

## Introduction

Self-organization plays an important role in nature – and more and more in technology [1–2]. Increasingly complex structures can evolve from using principles of self-organization in a bottom-up approach rather than from lithography-based top-down approaches. The key issue for intelligent self-assembly of complex structures is the design of local geometrically selective and site-selective interactions on the nanometer scale [3–6]. The more selective the interaction between the individual assembled components, the higher the complexity of the resulting structures that can be achieved.

One type of self-assembly is template-guided self-assembly, which plays an important role in biological processes relevant for biomineralization [7–12]. There are numerous approaches to harness and use this principle for artificial processes, which may be of great technological significance [13–15]. Recently, we reported the site-selective mineralization of a semiconductor material, zinc oxide (ZnO), on a chemically patterned surface [3]. ZnO thin films are of special interest since they can be used for different applications such as solar cells [16], biosensing devices [17] and others [18]. By using a nearly topographically flat (<1 nm roughness), but chemically patterned surface as a template, it was possible to guide the deposition – mainly by means of surface polarity. While deposition took place at sites with amino functionalization, no deposition was observed at locations with a fluorinated surface functionality. In this way, it was possible to guide the deposition using only chemical surface functionality with a topography-free, flat template.

In a next step towards template-controlled deposition, it would be desirable not only to predetermine the deposition sites by means of a chemical pattern template, but also the type of material deposited. In this way the properties of the deposited material are controlled depending on the surface functionality of the template. The properties that can be controlled by template functionality can be structural, topographical, electrical, mechanical, piezoelectrical, adhesive, tribological, catalytic activity [19] or properties connected with the granularity of the film [20–23]. Additionally, the reflectivity or light scattering properties may be controlled – the latter of which are highly relevant for the fields of optical data storage [24–25] and lithography (where increasingly smaller structures are sought, e.g., in the field of semiconductor nanolithography). Here, the copying of a given structure by self-templating may provide an alternative to conventional replication.

In this study, ZnO-containing films were prepared using chemical bath deposition. Two self-assembled monolayers (SAMs) with amino or fluorinated functionality were used to control the structure, and therefore, the roughness of the deposited film. A possible mechanism is presented that explains the influence of the template on the film formation.

## Results and Discussion

**Figure 1:**
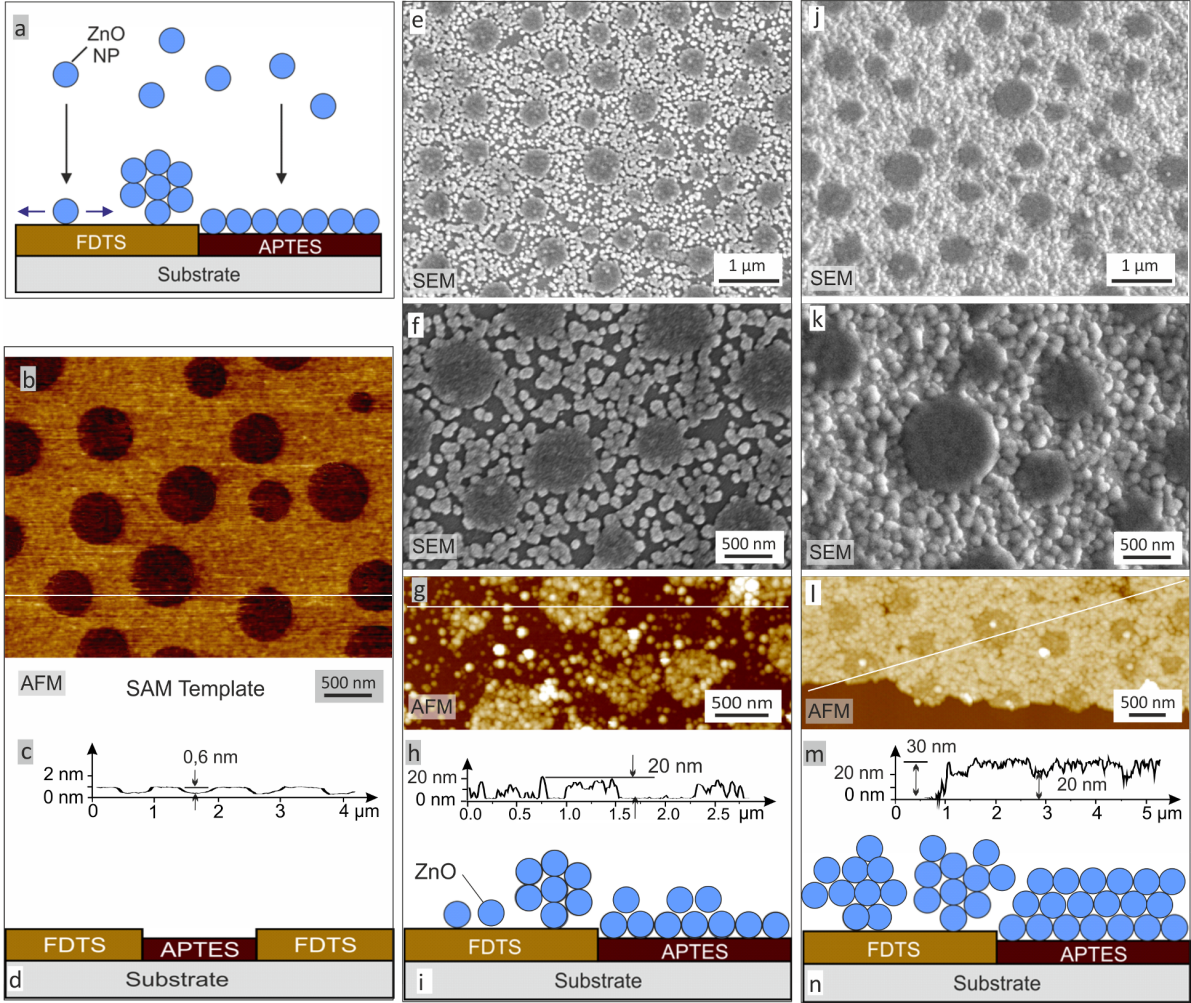
Self-assembly of ZnO-containing material on prepatterned substrates. (a) Schematic representation of the deposition mechanism. (b–d) The nonmineralized substrate shows the APTES islands (dark red) in the FDTS matrix (yellow). (e–i) SEM and AFM images show the deposited material on the templates. On the APTES, a smooth and compact film is formed, whereas on the FDTS agglomerates are deposited. With an increasing amount of deposited material, those agglomerates grow together (j–n) resulting in a rough surface for the final morphology.

Structured templates with polar 3-(aminopropyltriethoxy)-silane (APTES) and nonpolar 1H,1H,2H,2H-perfluorodecyl trichlorosilane (FDTS) areas were used for the deposition of nanostructured ZnO-containing films. Figure 1 shows atomic force microscopy (AFM) and scanning electron microscopy (SEM) images of the original templates and the resulting films together with schematic representations of the deposition mechanism (Figure 1a,d,i,n). The height difference between the two SAMs is 0.6 nm (Figure 1c). Analysis of the topographic images shows no significant difference in roughness between the different templates, as both surfaces exhibit an rms-roughness value of 0.1 nm. After mineralization, the APTES islands are covered by a homogenous, smooth film with an rms-roughness of 1 nm. AFM images show a granular structure that is not clearly visible in the corresponding SEM image. This might be due to the lower sensitivity to topographic features of the SEM.

Temperature has a significant influence on the deposition behavior. For higher temperatures, there is bulk precipitation and an inhomogenous film is formed. At lower temperatures, the growth rate is drastically reduced so that film formation is very slow. The reaction temperature of 70 °C is optimum for controlled deposition of the NPs.

In contrast to the situation on the APTES islands, on the FDTS matrix, large agglomerates with a diameter of 75 nm were deposited. Finally, this leads to a continuous and unperforated film with a roughness of 2.5 nm.

**Figure 2:**
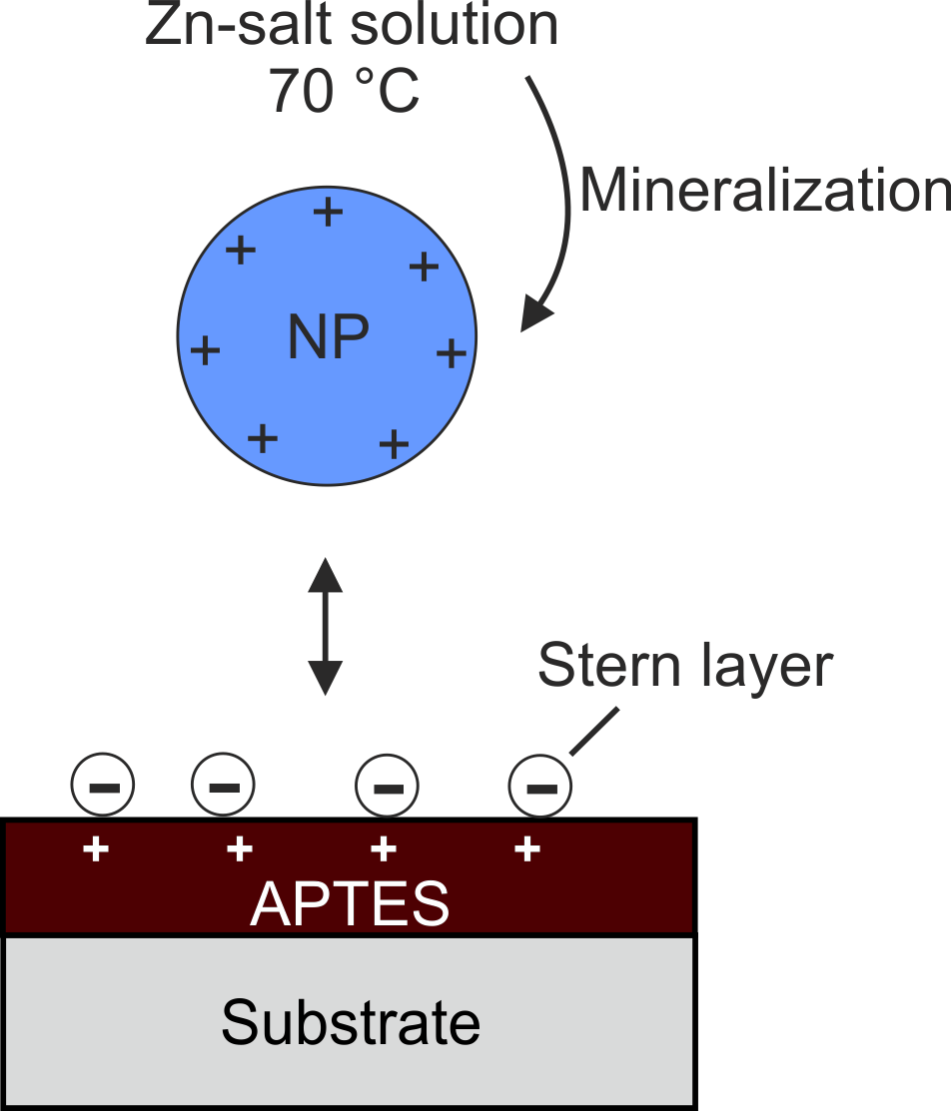
Deposition mechanism of mineralized ZnO nanoparticles on amino SAMs. The negative charges represent counterions attached to the positive surface charge (Stern layer) provided by protonated amino groups (–NH_3_^+^).

These ZnO-containing structures consist of particles formed in the deposition solution. These NPs grow in solution under the presence of histidine. Gerstel et al. [26–27] found that histidine controls NP growth and is incorporated in the resulting films. XRD measurements show that the deposited ZnO is X-ray-amorphous (data not shown). The investigation of the suspensions from the reaction solution by zeta potential measurements revealed that the particles possess a potential of +22.0 mV at pH 6.7 [26]. Since the pH of the reaction solution is around 5.3, the formed NPs are positively charged (Figure 2).

The zeta potential of the amino-functionalized SAM is charged slightly positive during the reaction [28–29] due to protonation of the amino groups (–NH_3_^+^) at this pH. Additionally, a Stern layer is present, which is formed by negatively charged counterions [29–30]. The particles in solution can interact with these anions and Coulomb forces lead to a strong binding to the surface (Figure 2). Furthermore, entropic forces, including counterion release forces, may contribute to an enhanced interaction. This leads to closer contact between the NPs and the template, providing a means to activate van der Waals short-range forces. Together these mechanisms lead to a homogenous film with a smooth surface in the APTES-functionalized holes (Figure 1).

The FDTS on the other hand is highly hydrophobic [31]. Electrostatic interactions with the particles are minimal compared to the deposition on APTES, where a homogenous and dense distribution of the surface charges leads to a high probability of interaction with particles. In the FDTS areas, small defects with low density can explain the presence of particles on the hydrophobic surface. During the template preparation process, APTES molecules may be deposited in these sites. The ZnO particles are attracted to these polar areas. Other particles are highly mobile due to the decreased interaction with the template. They can diffuse to the immobilized ones and decrease the interfacial energy by agglomeration. The result is a coarse granular structure that can be observed in SEM and AFM (Figure 1) on the FDTS regions of the substrate. When more and more material is deposited, those agglomerates can form a closed film, but with a significantly higher roughness compared to the films formed on the polar APTES-monolayer as shown in Figure 1j–n.

Forthcoming investigations will reveal if other properties such as piezoelectric activity, conductivity, optical or mechanical properties can also be controlled by the patterned surface chemistry of the substrate.

## Conclusion

Here we demonstrate the control of the structure and granularity of a growing film by means of a chemical functionality pattern of the substrate, where the chemical pattern acts as a template. A site-dependent granularity in mineralized ZnO-containing films is observed by self-assembly of nanoparticles during chemical bath deposition on patterned self-assembled monolayers. The influence of template regions of different polarities and surface energies on the deposition of thin ZnO-containing films was investigated. The positively charged amino-functionalized surface areas lead to a homogenous film with low roughness. The use of an uncharged hydrophobic SAM molecule (FDTS) supports the formation of coarse agglomerates with a higher roughness and irregular surface structure.

These findings open intriguing perspectives to control further properties that depend on film granularity such as optical, mechanical, piezoelectrical or tribological properties, by means of the chemical functionality pattern of a templating substrate – properties which, in turn, are key properties for nanodevices.

## Experimental

### 

#### Template preparation by polymer-blend lithography

Polymer solution: Polystyrene (PS, *M*_w_ = 96 kg/mol, PDI 1.04) and poly(methyl methacrylate) (PMMA, *M*_w_ = 9.59 kg/mol, PDI 1.05) were purchased from Polymer Standards Service GmbH and dissolved directly in methyl ethyl ketone (MEK, Aldrich). The mass ratio between PS and PMMA was 3:7 and the total concentration of the two polymers was 15 mg/mL.

Thin polymer-blend films were spin-coated at 1500 revolutions per minute (rpm) onto silicon substrates that were previously cleaned by CO_2_ snow-jet treatment (at least 20 s for a 2 × 2 cm substrate). The relative humidity, measured by a Testo 635 Hygrometer, was adjusted to 40–45% during the spin-coating process. For the adjustment of the humidity, a mixture of water-saturated and pure nitrogen were led into the spin-coating chamber (approximately 1 L volume) at a flow rate of approximately 40 standard cubic centimeters per minute (40 sccm).

#### Fabrication of SAM templates

After spin coating, the polymer films were treated with acetic acid where PMMA was selectively dissolved. The silicon samples were rinsed with the acid for 30 s and gently dried in a nitrogen flow. This procedure was repeated two times with fresh solvent. The fluorinated SAM was deposited from the gas phase: The samples were positioned face down at the lid of a desiccator containing two droplets of 1H,1H,2H,2H-perfluorodecyl trichlorosilane (Sigma-Aldrich) and evacuated to a pressure of 50 mbar. After 10–12 h in the desiccator, the samples were treated by CO_2_ snow-jet in order to remove the PS islands and to expose islands of bare SiO*_x_*-surface within the FDTS background. These islands were then back-filled by exposure to the vapor of 3-(aminopropyltriethoxy)-silane (Sigma-Aldrich). Further details and important parameters of the polymer-blend lithography process are described in [32]. The resulting pattern, consisting of amino-functionalized islands in a Teflon-like matrix (Figure 1b–d), was used as a template for the mineralization.

#### Mineralization experiment

All deposition solutions were freshly prepared prior to use to ensure clear starting solutions. Stock solutions of Zn(NO_3_)_2_∙6H_2_O (Sigma-Aldrich, ≥99.0%), hexamethylenetetramine (HMTA, Sigma-Aldrich, ≥99.5%) and L-histidine (Sigma-Aldrich, ≥99%) in Milli-Q water each at a concentration of 45 mM, and were prepared according to Gerstel et al. [26]. For the preparation of the mineralization solution, equal amounts of HMTA and histidine stock solutions were mixed. Afterwards, the zinc nitrate solution was added dropwise to obtain a ratio of [Zn^2+^]/[HMTA]/[His] of 1:1:1. The prestructured wafer was placed in 2 mL of the mineralization solution in a closed vessel and heated to 70 °C for 4 h. Several deposition experiments were performed and most yielded similar results; however, for some samples, no deposition was observed even after 4 h.

#### Characterization

Atomic force microscopy images were obtained with a commercial Dimension Icon system (Bruker) in tapping mode under ambient conditions. SAM templates were scanned under water in order to exclude the effect of meniscus forces of possible surface adsorbed water films on the topographic measurements.

Scanning electron micrographs were taken using a DSM 982 Gemini (Zeiss) at 3 kV and a working distance of 1–3 mm. To ensure conductivity, 0.2 nm of Pt/Pd (80:20) was sputtered onto the samples.
